# Genetic control of morphometric diversity in the maize shoot apical meristem

**DOI:** 10.1038/ncomms9974

**Published:** 2015-11-20

**Authors:** Samuel Leiboff, Xianran Li, Heng-Cheng Hu, Natalie Todt, Jinliang Yang, Xiao Li, Xiaoqing Yu, Gary J. Muehlbauer, Marja C. P. Timmermans, Jianming Yu, Patrick S. Schnable, Michael J. Scanlon

**Affiliations:** 1 Division of Plant Biology, Cornell University, Ithaca, New York 14850, USA; 2 Department of Agronomy, Iowa State University, Ames, Iowa 50010, USA; 3 Department of Agronomy and Plant Genetics, University of Minnesota, St Paul, Minnesota 55108, USA; 4 Cold Spring Harbor Laboratory, Cold Spring Harbor, New York 11724, USA

## Abstract

The maize shoot apical meristem (SAM) comprises a small pool of stem cells that generate all above-ground organs. Although mutational studies have identified genetic networks regulating SAM function, little is known about SAM morphological variation in natural populations. Here we report the use of high-throughput image processing to capture rich SAM size variation within a diverse maize inbred panel. We demonstrate correlations between seedling SAM size and agronomically important adult traits such as flowering time, stem size and leaf node number. Combining SAM phenotypes with 1.2 million single nucleotide polymorphisms (SNPs) via genome-wide association study reveals unexpected SAM morphology candidate genes. Analyses of candidate genes implicated in hormone transport, cell division and cell size confirm correlations between SAM morphology and trait-associated SNP alleles. Our data illustrate that the microscopic seedling SAM is predictive of adult phenotypes and that SAM morphometric variation is associated with genes not previously predicted to regulate SAM size.

## Abstract (web summary)

In plants, the shoot apical meristem generates all of the above ground organs and meristem morphology may predict important agricultural traits. Here Leiboff *et al*. use high throughput phenotyping and a genome-wide association study to uncover genes associated with variation in maize meristem size.

## Graphical abstract

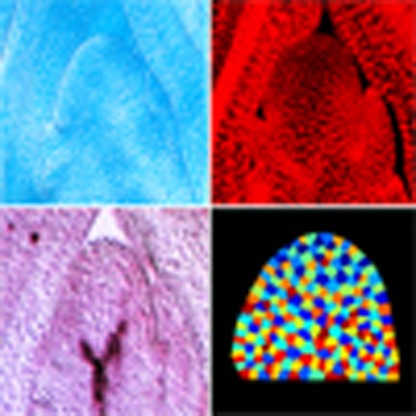

Plants maintain populations of pluripotent stem cells called shoot apical meristems (SAMs) throughout their lifetime. Shoot meristems function to generate morphologically complex body plans by the coordinated activities of stem cell maintenance to sustain the SAM, and organogenesis of leaves and branches in a phyllotactic pattern^1^. These dual SAM functions determine the number and position of all lateral organs that make up the plant shoot. Although microscopic in size, correlations of seedling SAM morphology and adult plant phenotypes may render the vegetative SAM predictive of agronomically important plant traits^2^.

Decades of genetic research have delineated a complex, interactive network of transcription factors, hormonal signals, epigenetic marks, metabolites and biophysical forces that contribute to the regulation of SAM function^3^,^4^,^5^,^6^,^7^,^8^. Single-gene mutations within these SAM genetic networks can alter the morphology of both the shoot meristem and the plant^9^,^10^, revealing that SAM structure and function are intimately linked. Although these studies identified a number of genes required for SAM function, little is known about the genetic control of SAM morphological variation in large natural populations or in diverse breeding stocks. QTL analyses of bi-parental populations have shown that differences in SAM morphology may involve loci not previously identified via single-gene mutations^2^,^11^.

In contrast to QTL analyses, genome-wide association studies (GWAS) exploit historical recombination events and linkage disequilibrium to dissect the genetic architecture of quantitative traits. The abundant polymorphism and relatively low linkage disequilibrium present in the model crop plant maize (*Zea mays* subsp. *mays* L.), when coupled with exhaustive genotypic surveys and innovative statistical analyses, have increased the precision and the power to identify genic associations for multiple maize traits^12^. Thus, further interrogation of the genetic architecture of SAM morphology among many diverse genetic stocks may reveal novel regulators of SAM function, which have not been highlighted by single-gene mutations or QTL analyses of bi-parental populations.

To date, the majority of maize GWAS have analysed the genetic basis of macroscopic or biochemical phenotypes in adult plants^12^,^13^,^14^. Although a few studies in other systems have examined microscopic phenotypes^15^,^16^, no GWAS in maize has utilized phenotypes collected at a microscopic scale. Here we report the first application of GWAS to study the genetic architecture of maize SAM morphology, a microscopic phenotype that poses unique challenges for quantitative analysis. Applying a high-throughput imaging pipeline to a diverse panel of 369 maize inbred lines, we detect extensive SAM morphometric variation. Significant correlations are identified between the microscopic SAM and several adult phenotypes, including flowering time, stem width and leaf node number. These findings demonstrate that the morphology of the seedling SAM is predictive of agronomically important adult plant traits. Utilizing a 1.2 million single nucleotide polymorphism (SNP) data set that combined RNAseq-generated and previously published available genotypes, we identify candidate genes associated with SAM morphological variation. Although the majority of these GWAS-derived SAM candidate genes have not been previously implicated in studies of SAM structure, subsequent analyses of candidate genes with putative functions in hormone transport, cell division and cell expansion support their predicted contributions to maize SAM morphological diversity.

## Results

### The maize SAM morphospace correlates with adult plant traits

Although several groups have documented differences in SAM morphology among common maize inbred varieties^2^,^11^,^17^, to date no studies have summarized the diversity of shapes and sizes that populate the maize SAM morphospace. We adapted a high-throughput histological clearing technique^11^,^18^ to image a panel of 369 diverse maize inbred varieties that represents more than 80% of the genetic diversity within *Z. mays* subsp. *mays* L^19^,^20^. We hypothesized that this panel would likewise capture much of the natural variation in SAM microphenotypes.

**Figure 1:**
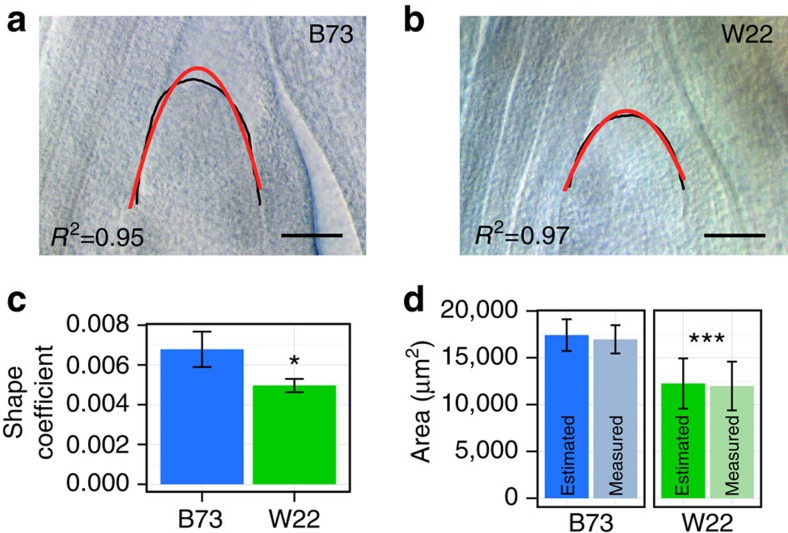
Parabolic models of the maize SAM allow rapid assessment of morphology. (**a**,**b**) Inbreds B73 and W22 have SAM morphology (black) closely approximated by a parabolic model (red). (**c**) Parabolic SAM model coefficients between inbreds (*n*=10) are significantly distinct, Student's *t*-test, *P*=0.013 (**d**) SAM area calculations from model-derived estimates, ‘Estimated' and direct image-processed measures, ‘Measured' are not significantly different within a genotype, but significantly different between genotypes. Two-way ANOVA—factor, genotype: *P*=0.000325; factor, measurement technique: *P*=0.750547; interaction, genotype and technique: *P*=0.939353. Scale bars, 100 μm. Error bars, 95% CI.

We modelled the SAM as a paraboloid, a geometric shape that facilitates estimations of multiple measures such as volume, surface area, arc length and curvature, all of which can be calculated from just two discrete measurements, SAM height and SAM radius^21^,^22^. To test the efficacy of this parabolic model, we analysed two maize inbred lines (B73 and W22) with demonstrated differences in SAM size (Fig. 1)^17^. Modelling the SAM as a paraboloid identified statistically significant differences between inbreds in shape-determining model coefficients (Fig. 1a–c). In a comparison of direct image processing and parabolic modelling of SAM microphenotypes, we found no statistical difference between measurement methods, yet the ability to differentiate genotypes was maintained (Fig. 1d). We incorporated this parabolic model into our image-processing pipeline to quickly generate many SAM microphenotypes from rudimentary primary measurements (Supplementary Data 1).

**Figure 2:**
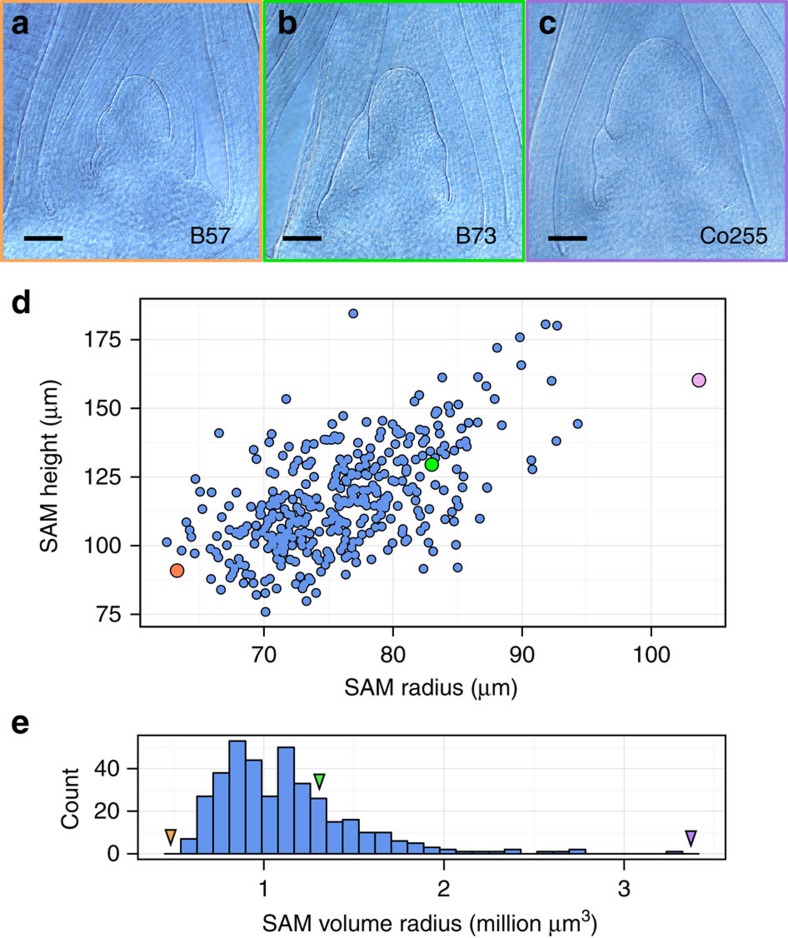
Extreme variation in maize SAM morphology. (**a**–**c**) Examples of small (**a**), intermediate (**b**) and large (**c**) SAM lines. (**d**) BLUPs calculated for SAM height and radius, (*n*=4). (**e**) SAM volume estimated by parabolic model of SAM shape. Points/arrows highlight small, intermediate and large accessions, B57 (orange), B73 (green) and Co255 (purple), respectively. Scale bars, 100 μm.

Across four biological replicates, we identified rich diversity in SAM morphology within our panel of 369 maize inbreds (Fig. 2, Supplementary Data 2). Figure 2d portrays the maize SAM morphospace, plotted as SAM radius versus height; small, intermediate and large size categories of maize SAMs were identified. The SAM from the inbred line B73, from which the reference maize genome was obtained^23^, occupies the centre of our morphospace (Fig. 2a–d). Modelling the SAM as a paraboloid enabled facile estimations of SAM volume (Fig. 2e).

In comparisons of microscopic SAM seedling phenotypes to agronomically important adult plant traits (Supplementary Data 3), we identified modest but significant correlations between seedling SAM volume and height to primary ear (Pearson's *r*=−0.18, Fisher transformation *P*=9.871e−04), days to anthesis (Pearson's *r*=−0.33, Fisher transformation *P*=6.743e−10), leaf node number (Pearson's *r*=−0.21, Fisher transformation *P*=8.922e−05) and stem diameter above the primary ear (Pearson's *r*=−0.13, Fisher transformation *P*=0.01238) (Supplementary Fig. 1)^24^.

### GWAS of maize SAM volume

To better understand the genetic architecture controlling maize SAM morphology, we used GWAS to identify loci correlated with SAM microphenotypes within our diverse maize inbred panel. SAM volume was found to have a favourable entry mean heritability, or repeatability of 0.84, and its calculation captures variation contributed by multiple SAM microphenotypes (Supplementary Fig. 2, Methods). We therefore focused our analyses on SAM volume.

**Figure 3:**
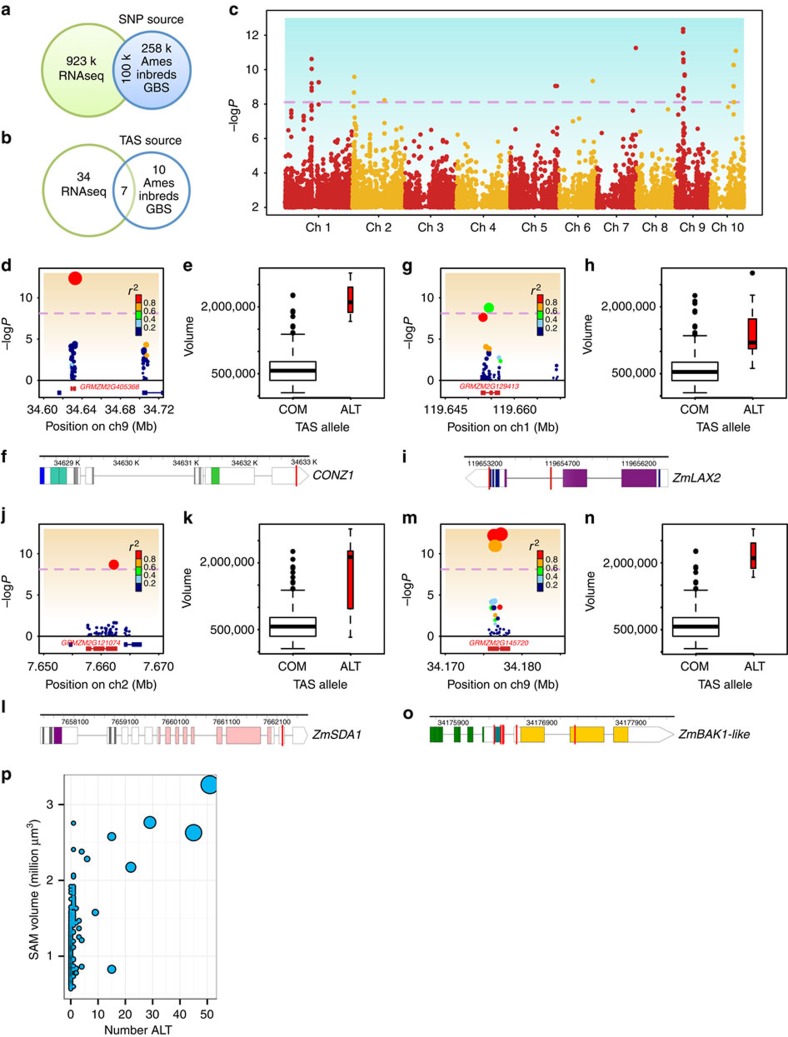
GWAS of maize SAM volume. (**a**) SNP genotyping matrix generated by public sequences (Ames Inbreds) and RNAseq-derived SNPs. (**b**) Trait-associated SNPs (TASs) from both SNP sources. (**c**) Manhattan plot of GWAS showing 51 TASs with −log*P*>8.11, dotted line. Candidate genes with predicted developmental functions (**d**–**o**). (**d**,**g**,**j**,**m**) Regional plots show significant SNPs and linkage disequilibrium (colour scale) around candidate genes (red). (**e**,**h**,**k**,**n**) SAM volume for common (COM) and alternative (ALT) TAS alleles. Width scales with number of inbreds. (**f**,**i**,**l**,**o**) Gene models—coloured rectangles indicate predicted protein domains. Location of TAS, red line. (**d**–**f**) GRMZM2G405368, *Constans-like 1* (*CONZ1*). (**g**–**i**) GRMZM2G129413, *Like Auxin-Resistant 2 (ZmLAX2*). (**j**–**l**) GRMZM2G121074, *severe depolymerization of actin1* (*ZmSDA1*). (m–o) GRMZM2G145720, *BRASSINOSTEROID INSENSITIVE 1-associated receptor kinase 1 like* (*ZmBAK1-like*). (**p**) Number of ALT TAS alleles for inbred lines of different SAM volume.

Using RNAseq data obtained from SAM-enriched apex tissue, we generated 923,000 novel SNPs from our maize inbred panel. Additional 358,000 SNPs called from the Ames US Inbreds public data set generated a combined genotyping matrix of more than 1.2 million high quality SNPs (Fig. 3a)^25^,^26^. We used a unified mixed-model approach to associate SAM volume with SNPs from our genotyping matrix, accounting for kinship and population structure within the panel^27^. Fifty-one trait-associated SNPs (TAS) that surpassed a stringent (*α*=0.01) Bonferroni-correction threshold of −log*P*>8.11 were detected. Thirty-four TAS were unique to RNAseq-generated SNPs, while only seven TAS were found in both SNP data sets (Fig. 3b). Forty-eight TAS were within 100 kb of 23 unique candidate genes, with the majority of TAS (44/48) in predicted coding regions themselves (Fig. 3c–o, Supplementary Data 4). This bias towards coding regions is in accordance with a previous report of GWAS conducted using SNPs generated from RNAseq data^26^.

In each of the 51 TAS, the common allele (COM) had a frequency above 91% (Supplementary Data 5). For all but one TAS, the B73 reference sequence was the COM. The total number of TAS alternate alleles (ALT) identified in an individual was moderately correlated with SAM volume (Pearson's *r*=0.50, Fisher transformation *P*<2.22e−16); inbreds with the largest SAMs were more frequently ALT at multiple TAS (Fig. 3p). We selected four candidate genes with especially interesting predicted developmental functions for analyses of the contribution of ALT alleles to SAM shape and size.

### SAM morphology-associated genes

We detected one TAS within the 3′ untranslated region of GRMZM2G405368, *Constans-like 1* (*CONZ1*) (Fig. 3c). *CONZ1* exhibits diurnal transcript fluctuations and is associated with flowering time^28^. Within our maize inbred panel, we found a significant, moderate/weak negative correlation between SAM volume and days to anthesis (DTA; Pearson's *r*=−0.33, Fisher transformation *P*=6.743e−10)^24^. Although flowering time data is available for just one of the four *CONZ1-ALT* lines, the DTA value for Co255 falls within the upper quartile of this inbred panel. Morphological examination of sampled SAMs revealed active production of leaf primordia (Figs 1a,b and 2a–c), verifying that the SAMs assayed in our data set had not undergone the transition from vegetative to inflorescence-staged shoot meristems^4^. Furthermore, neither CONZ1 nor any SAM morphology-associated candidate genes identified in our study have been implicated in prior GWAS of maize flowering time^14^,^24^.

We detected two TASs within the 2nd intron and 3rd exon of GRMZM2G129413, which appear as one allele in our panel (Fig. 3d). The ALT form of the 3rd exon TAS is expected to render an amino acid change from histidine to asparagine near a predicted low-complexity protein domain. The closest *Arabidopsis thaliana* homologue to GRMZM2G129413 is *Like Auxin-Resistant 2* (*LAX2*), a predicted auxin influx protein that is expressed within developing vasculature and may modulate auxin flow dynamics^29^,^30^,^31^.

**Figure 4:**
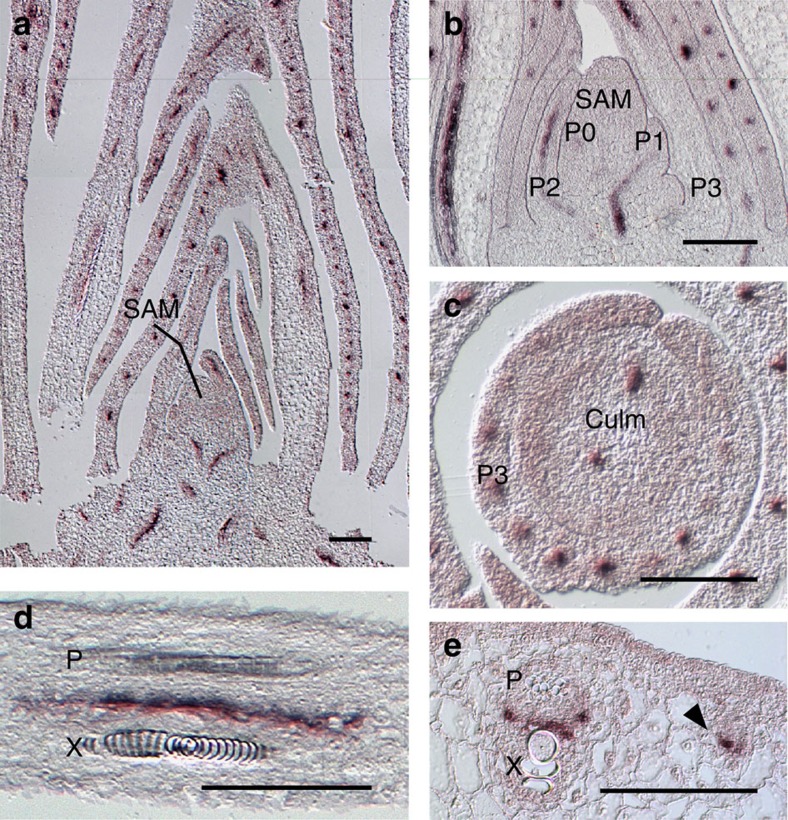
Transcript accumulation pattern of *ZmLAX2*. (**a**,**b**) *In situ* hybridization of 14-day-old maize seedlings (inbred B73) reveals *ZmLAX2* transcript localization in the vascular traces of leaf primordia. (**c**) Punctate provascular accumulation found in transverse of culm below the SAM. (**d**,**e**) Procambial cells between mature phloem, ‘P', and xylem, ‘X', show accumulation in longitudinal and transverse of P7 primordium. (**e**) Arrow denotes procambial expression in minor vein prior to xylem and phloem differentiation. Scale bars, 100 μm.

*In situ* hybridization of B73 maize seedling apices (*n*=20) with a probe specific to *ZmLAX2* shows a strong provascular expression pattern within leaf primordia and in the developing stem (Fig. 4a–c). Expression is not detected in differentiated xylem or phloem cells, but is restricted to the procambium, undifferentiated cells located between the xylem and phloem poles (Fig. 4d,e). Due to the three-dimensional arrangement of plant vasculature, single longitudinal sections do not capture entire vascular traces. To address this issue, we aligned and compiled our *ZmLAX2 in situ* hybridization data from several serial sections from 10 additional inbred lines selected to reflect various SAM sizes and *ZmLAX2* genotypes to reconstruct the native expression pattern.

**Figure 5:**
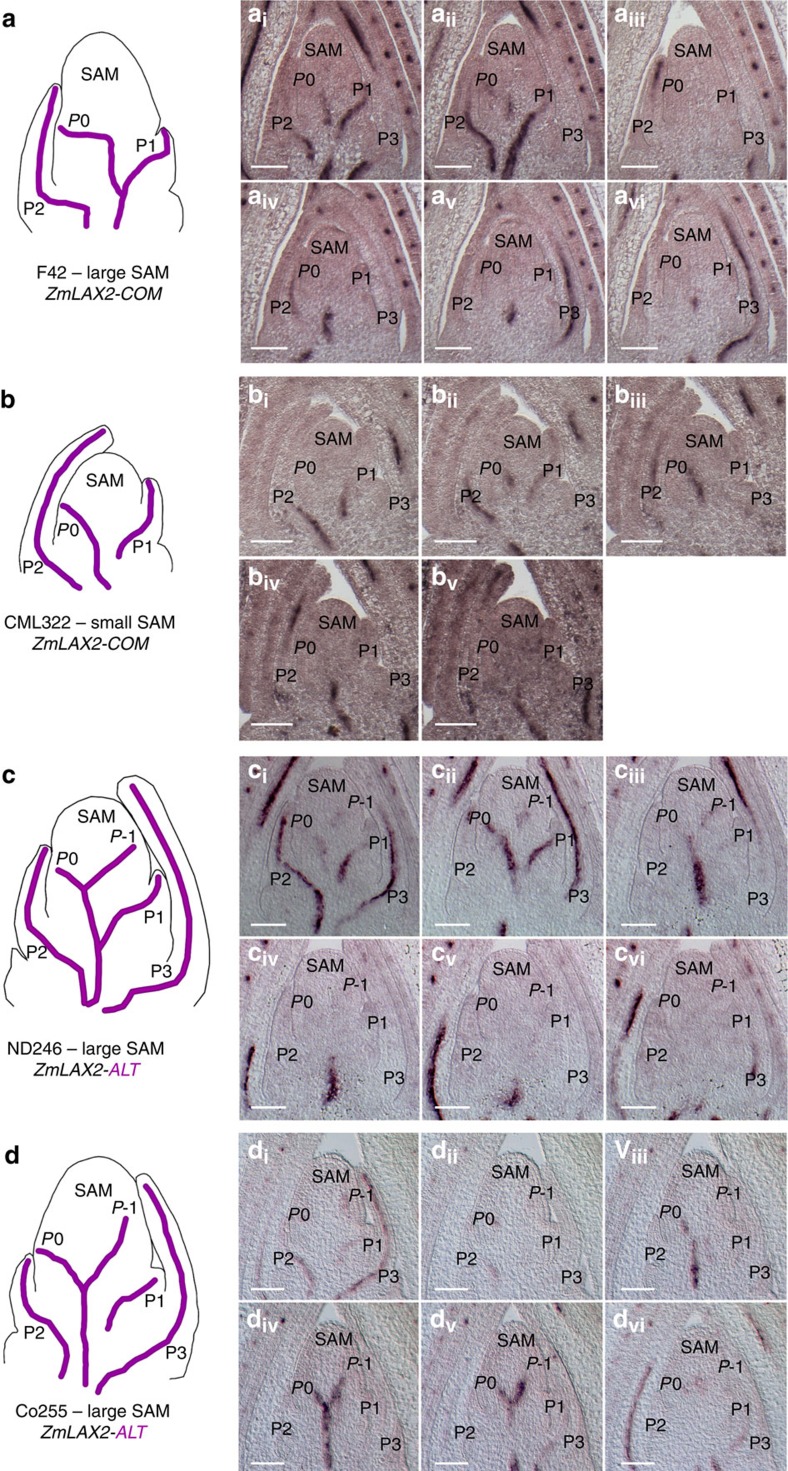
Spatiotemporal patterns of *ZmLAX2* transcript differ by common or alternate *ZmLAX2* TAS genotype. Serial section compilations from large (**a**) and small (**b**) SAM *ZmLAX2-COM* (common) lines show transcript accumulation in P0 and later primordia, P1–P3. Serial sections (a_i_–a_vi_, b_i_–b_v_). Large SAM *ZmLAX2-ALT* (alternate) lines (**c**,**d**) exhibit additional transcript accumulation above the P0, in the position of the next anticipated primoridium, P-1. Serial sections (c_i_–c_vi_, d_i_–d_vi_). Scale bars, 100 μm.

We detected spatiotemporal variation in *ZmLAX2* transcripts correlated with the *ZmLAX2* TAS genotype (Fig. 5). Leaves are designated according to plastochron number, which specifies the relative time elapsed since initiation from the SAM, such that the newly initiated leaf is termed P1 and the next incipient primordium is designated P0 (ref. 32). In four large SAM *ZmLAX2*-*COM* lines, transcript accumulation was detected in the P0 and in older leaf primordia (Fig. 5a). Similarly, four small SAM *ZmLAX2*-*COM* lines examined exhibited *ZmLAX2* transcript accumulation in the P0 and older primordia (Fig. 5b). In contrast, the large SAM *ZmLAX2-ALT* lines ND246 and Co255 displayed transcript accumulation in the P0 and older leaf primordia, as well as on the flank of the SAM opposite the P0 (Fig. 5c,d). This unique expression pattern extends into the SAM towards the predicted location of the yet-to-be-specified incipient primordium, designated P-1. Note that the accumulation of *ZmLAX2* transcript in P-1 primordia can been seen in apices with relatively larger, flanking P1 and P2 primordia (Fig. 5c), as well as shoot apices with smaller P1 and P2 primordia (Fig. 5d). Thus, the observed accumulation of *ZmLAX2* transcript in P-1 primordia in large SAMs containing the *ZmLAX2-ALT* allele is not correlated with plastochron index and this expression pattern is not an artifact of relative developmental staging between plastochrons.

We detected one TAS within the 14th exon of GRMZM2G121074 that is predicted to cause a synonymous codon change in the *ZmSDA1-ALT* allele. GRMZM2G121074 is the closest maize homologue of *severe depolymerization of actin* (*SDA1*), a highly conserved gene required for cellular G1 phase transition and mitotic timing in *Saccharomyces cerevisiae*^33^,^34^.

**Figure 6:**
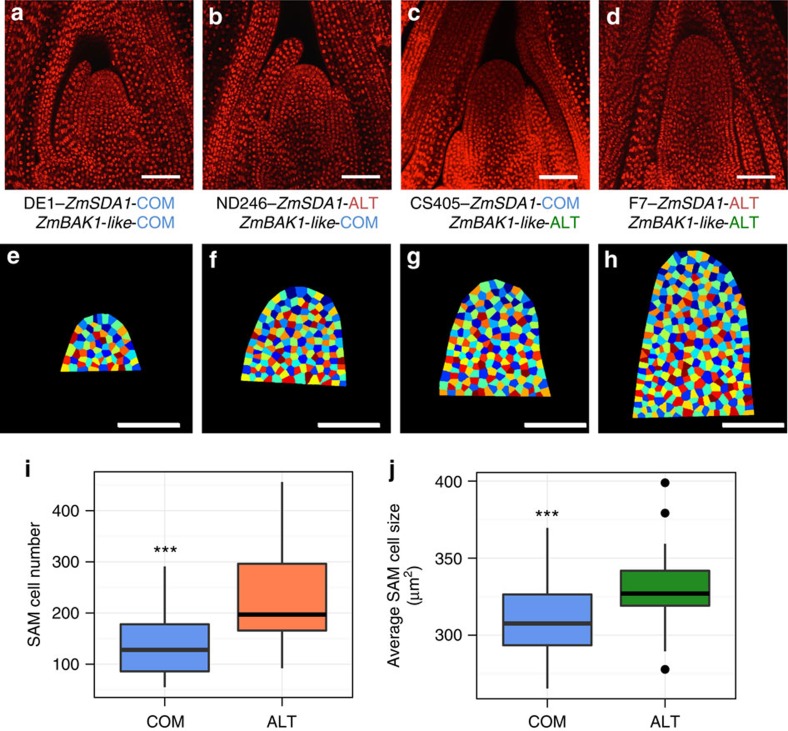
SAM cell number and cell size are correlated with *ZmSDA1-ALT*and *ZmBAK1-like-ALT* alleles. Automated cell segmentation of SAM images from *ZmSDA1-ALT* (*n*=3) and *ZmBAK1-like-ALT* (*n*=5) lines compared with large (*n*=3), intermediate (*n*=3) and small (*n*=3) common (COM) TAS allele lines. (**a**–**d**) SAM images collected by confocal microscopy. (**e**–**h**) Cell segmentation of SAM images identifies nuclei and divides space between them into a lattice of cells, used to determine cell number and cell size. (**i**) *ZmSDA1-ALT* lines (**b**,**f**) have increased SAM cell number, compared with *ZmSDA1-COM* lines (**a**,**e**), independent from SAM volume effects: two-way ANOVA—factor, *ZmSDA1*: *P*=4.37e−14; Factor, SAM volume: *P*=2.98e−15; Interaction, *ZmSDA1* and SAM volume: *P*=0.731. (**j**) *ZmBAK1-like-ALT* lines (**c**,**g**) have increased average SAM cell size, compared with *ZmBAK1-like-COM* lines (**a**,**e**), while SAM volume does not have a significant effect: two-way ANOVA—factor, ZmBAK1-like: *P*=0.000137; factor, SAM volume: *P*=0.516722; interaction, ZmBAK1-like and SAM volume: *P*=0.141583. Scale bars, 100 μm. ***P-value<0.001.

We processed images from a subset of inbred lines treated with a Kasten's fluorescent Feulgen stain to test whether *ZmSDA1* genotype is correlated with differences in cell number (Fig. 6)^35^,^36^,^37^. Images from 3 *ZmSDA1-ALT* lines and 11 randomly chosen *ZmSDA1-COM* lines with small, intermediate and large maize SAMs were examined in 3 biological replicates (Fig. 6a–h). *ZmSDA1-ALT* lines exhibited a statistically significant increase in SAM cell number (SCN) compared with *ZmSDA1-COM* lines (Fig. 6i). Modelling SCN as the product of *ZmSDA1* genotype and SAM volume in a two-way ANOVA showed that *ZmSDA1* genotype and SAM volume are both significant predictive factors of SCN, and predictions of SCN are independent of the interaction between *ZmSDA1* genotype and SAM volume.

We detected four TASs within the 4th exon, one TAS within the 5th exon and one TAS within the 6th exon of GRMZM2G145720, which appear as one allele in our panel. GRMZM2G145720 is a leucine-rich repeat receptor-like protein kinase gene homologous to the *Oryza sativa* gene *BRASSINOSTEROID INSENSITIVE 1-associated receptor kinase 1* (*OsBAK1*) (maizegdb.org). The ALT allele of *ZmBAK1-like* encodes two expected amino acid changes flanking a predicted transmembrane domain, lysine to arginine and arginine to threonine, respectively. In *O. sativa*, *BAK1* participates in brassinosteroid-dependent cell expansion^38^. We therefore tested if cell size is affected in *ZmBAK1-like-ALT* lines.

As above, we processed SAM images from a subset of inbred lines to test the correlation of *ZmBAK1-like-ALT* and cell size (Fig. 6)^35^,^36^,^37^. Images from five *ZmBAK1-like-ALT* lines and nine *ZmBAK1-like-COM* inbred lines representing small, intermediate and large SAM size categories, were examined in three biological replicates (Fig. 6a–h). *ZmBAK1-like-ALT* lines exhibit a statistically significant increase in average SAM cell size (ASCS) compared with *ZmBAK1-like-COM* lines (Fig. 6j). Modelling ASCS as the product of genotype and SAM volume in a two-way ANOVA showed that SAM volume was insignificant in predicting ASCS; however, the *ZmBAK1-like-ALT* allele was a significant predictive factor for ASCS.

## Discussion

Previous studies of maize seedling SAM shape and size diversity have been limited to a small number of inbred varieties^2^,^11^,^17^. By approximating SAM shape with parabolic models, we were able to survey morphometric diversity in 369 maize inbred lines. GWAS of microscopic phenotypes such as macular degeneration in the human eye and root meristem size in the model plant *A. thaliana* identified a small number of statistically significant genotype–phenotype associations^15^,^16^. The high repeatability, or entry mean heritability, of SAM volume combined with our dense genotyping matrix of 1.2 million SNPs in a mixed-model approach allowed us to identify 51 TAS with a high stringency Bonferroni correction, *α*=0.01. Previous reports of microphenotype GWAS used molecular developmental strategies to support candidate loci^15^,^16^. Likewise, we used a variety of molecular developmental techniques to characterize a small number of SAM morphology candidate genes.

Using a high-throughput image-processing pipeline to generate SAM morphological data for GWAS of 369 maize inbred lines, we identified candidate genes involved in intraspecific SAM morphological variation. Studies of natural variation in plants and animals have found that biologically significant changes are often linked to polymorphisms in non-genic regulatory regions that may contribute to the evolution of novel expression patterns^39^,^40^,^41^,^42^. In contrast with this trend, the majority of our GWAS-identified TASs are found within predicted gene-coding regions. However, because 77% of SNPs from our genotyping matrix were generated by RNAseq analysis, we expect a bias towards the identification of genic polymorphisms by GWAS (Fig. 3b)^26^. Although several of our gene candidates have TASs within coding regions, and some ALT alleles encode for predicted amino acid changes that may alter protein function, further validation involving reverse genetics or fine-mapping of advanced introgression lines is required to confirm any potentially functional nucleotide polymorphisms. TASs identified in our analysis may be markers of causative changes in flanking regulatory regions, for which we have not identified polymorphic SNPs^26^,^41^. Nevertheless our data provides additional evidence that SNPs generated by RNAseq analysis can be used to generate a dense genotyping matrix for GWAS, allowing for high-resolution, single-gene associations^26^.

Our data agree with previous reports that correlate large SAM size with early flowering (decreased days to anthesis) phenotypes^2^, and our data expand this correlation to a markedly larger panel of inbred maize varieties. Previous research has shown that SAM size increases throughout vegetative development^2^,^43^,^44^, whereafter the SAM transforms into the male inflorescence meristem. Morphological evidence showing P1 and P0 leaf primordia arising from the periphery of all the samples examined in this study (Figs 1a,b and 2a–c) confirm that these SAMs are indeed vegetative shoot meristems and have not transformed into male inflorescences^4^. The significant correlation between large SAM size and early flowering suggests that large SAM lines undergo reproductive phase change earlier than small SAM lines. However, our SAM size GWAS did not detect genes previously implicated in regulation of flowering time^14^; in contrast, we find that natural variation in SAM size and flowering time are associated with separate genes.

Significant negative correlations between large SAM volume and plant height at the primary ear are likely to reflect the early flowering time of large SAM lines simply because large SAM lines terminate vegetative growth earlier in the season. We also detected a negative correlation between SAM size and leaf node number, a proxy for total leaf number, which would likewise be expected for plants that flower earlier in the growing season and therefore produce fewer leaves and stems. Significant correlations were likewise discovered between SAM size and stem diameter, which is an important factor in lodging resistance and damage from stem boring insects^45^,^46^. Such a correlation in seedling SAM size and adult stem size is quite remarkable, considering there is an ∼800-fold increase in size between the average SAM radius and the average stem diameter for the 369 lines in our study. Internode stem diameter and length are inversely correlated in maize and other grasses^47^,^48^, such that internode width decreases markedly in upper (younger) nodes as internode length increases. Our data suggest that the relationship between SAM size and stem diameter is driven by SAM height, whereas SAM radius is insignificant in explaining the correlation (Supplementary Fig. 1). Conversely, the relationship between SAM size and plant height to the primary ear is driven by SAM radius and not SAM height (Supplementary Fig. 1). We expect that these two relationships represent an allometric trade-off between plant height and stem diameter, separated into discrete internodes, that is established within the SAM. At the same internode, increased SAM height leads to decreased stem diameter and increased SAM radius leads to decreased plant height.

Although statistically significant, these correlations are moderate. Nonetheless, the data suggest that the stem cell population housed in the diminutive, microscopic maize seedling SAM is predictive of several impactful adult agricultural traits, despite substantial intervening development and growth.

This study uncovered 23 candidate genes associated with SAM size and shape. Notably, our GWAS did not detect any SAM master regulatory genes previously identified by mutational analyses, corroborating the results of previous QTL analyses of maize SAM morphology^2^,^11^. A successful GWAS ultimately links phenotypic variation with allelic polymorphisms. As such, our GWAS would fail to identify SAM master regulators if these genes were fixed in our population, perhaps due to strong purifying selective pressure for SAM function as observed in some species^49^. However, our genotyping matrix includes ample polymorphisms within the coding sequences of multiple SAM master regulatory genes (Supplementary Data 6). For example, after filtering and quality control, 118 SNPs were identified in the SAM maintenance gene *knotted1* (*kn1*)^50^ and 12 SNPs were found in the SAM size regulator *aberrant phyllotaxy1* (*abph1*)^10^, although SNPs in neither gene were significantly associated with SAM volume. Likewise, although 23 SNPs were identified in the leucine-rich repeat receptor-like, *faciated ear2* (*fea2*)^51^, significant associations were not detected between SAM volume and *fea2* SNPs by GWAS. Loss of *fea2*, a putative CLAVATA2 orthologue, dramatically affects the shape and size of the maize inflorescence meristem^51^, and natural variation in the regulation of *fea2* was shown to underlie ear morphological variation between maize inbreds B73 and Mo17 (ref. 52).

Notably, our data suggest that either known SAM master regulatory genes do not make major contributions to natural SAM morphometric variation, or else these contributions are not detectable in our experiment. Instead, our data suggest that SAM morphometric variation in natural populations and diverse breeding stocks is more likely attributed to allelic variation in genes regulating cell expansion and cell division (Fig. 6) as opposed to genes required for shoot meristem maintenance, stem cell indeterminacy or organ initiation. With additional investigation into potential developmental molecular mechanisms, the gene candidates identified in this study may provide novel insights into the regulation of SAM function.

This study revealed that allelic variants of *ZmLAX2*, a predicted member of the AUX/LAX family of auxin influx proteins^30^, are associated with SAM morphometric variation. Auxin canalization within the SAM is required for phyllotactic patterning and lateral organogenesis^53^,^54^,^55^. Canalization is established by the combined cellular efflux of PIN family proteins and auxin influx of AUX/LAX family proteins^53^,^56^,^57^. Cellular localization experiments and models of auxin flux dynamics both suggest that the mutually antagonistic functions of AUX/LAX and PIN proteins are localized to provascular traces that mark the developing leaf primordium (P0)^53^,^56^,^58^.

*In situ* hybridization reveals that *ZmLAX2* transcript accumulation coincides with previously described patterns of PIN localization in the developing leaf primordium (P0)^59^, suggesting that AUX/LAX protein family function has been conserved in maize. Interestingly, *ZmLAX2-ALT* inbred lines with large SAM phenotypes exhibit transcript accumulation in the developing leaf primordium (P0), as well as the yet-to-be-elaborated leaf primordium (P-1). This unique spatiotemporal expression pattern suggests that *ZmLAX2* transcript accumulation occurs prior to previously documented markers of vascular trace formation in *ZmLAX2-ALT* lines^59^,^60^. Because AUX/LAX influx functions are known antagonists of auxin canalization^53^ and NPA-mediated inhibition of auxin transport/canalization dramatically increases SAM size^61^, increased SAM size identified in *ZmLAX2-ALT* inbred lines may result from expanded or developmentally hastened expression of AUX/LAX family genes in the maize SAM.

## Methods

### Plant growth and tissue harvest

Plants for all experiments were grown under standard conditions with 10-hr day cycles in Percival A100 growth chambers (Percival Scientific, Perry, IA) planted in 98-well trays with all edge positions filled with inbred B73. Soil media was a 1:1 mixture of Turface MVP (PROFILE Products LLC, Buffalo Grove, IL) and LM111 (Lambert Peat Moss, Qc, Canada). All plants were harvested 14 days after planting and quickly trimmed to small SAM-containing tissue cassettes and fixed in FAA (3.7% formalin, 5% acetic acid and 50% ethanol in water) on ice, overnight.

For initial modelling, 10 kernels from inbred B73 and 10 kernels from inbred W22 were planted as above. To map the maize SAM morphospace, kernels from 384 inbred varieties (Supplementary Data 1) were planted in randomized positions in 4 biological replicates. For RNA *in situ* hybridization, 10 kernels from select lines were grown as above in 2 biological replicates. To estimate SAM cell count and ASCS, 4 kernels from 14 inbred varieties were planted with 3 biological replicates: 3 ZmSDA1-ALT lines and 5 ZmBAK1-like1 ALT lines with remaining lines randomly chosen to equally represent the lower quartile (small), middle quartiles (intermediate) and upper quartile (large) of SAM volume with COM from *ZmSDA1* and *ZmBAK1-like1*.

### SAM tissue preparation and imaging

For differential internal contrast (DIC) imaging of SAMs, FAA-fixed 14-day-old seedling tissue was dehydrated in an ethanol solution series and cleared overnight with methyl salicylate as used in the studies by Vollbrecht *et al*.^17^ and Thompson *et al*.^2^ Cleared tissue was imaged with Nomarski optics on an Axio Imager.Z10 (Carl Zeiss Microscopy, LLC, Thornwood, NY) with an AxioCam MRc5 camera. We captured near-median longitudinal optical sections using primordia appearance and SAM apex contours as morphological cues. Images are available at MaizeGDB (http://maizegdb.org).

For fluorescent staining of SAM nuclei, FAA-fixed 14-day-old seedling tissue was treated with Kasten's Feuglen stain as described in the studies by Ruzin^36^ and Kasten^37^: fully hydrated tissue was digested with 1 N hydrochloric acid overnight then reacted with a solution of safranin-O (safO) incubated with potassium metabisulfite and hydrochloric acid. After a brief destain, samples were dehydrated and cleared with methyl salicylate. Images were collected with a Leica TCS-SP5 (Leica Microsystems Exton, PA, USA) using an argon ion laser (488 nm). SafO-stained samples had a broad, low background emission spectrum (580–650 nm). Single optical sections were selected at near-median longitudinal planes. Images were processed using Leica LAS-AF software (version 2.6.0) prior to analysis.

### Image processing

For parabolic modelling of SAMs, DIC images from 14-day-old seedlings of inbred B73 (*n*=5) and inbred W22 (*n*=5) were processed using ImageJ^62^. To test the efficacy of a parabolic model of SAM curvature, custom macros were used to collect and export a traced SAM contour. Splines were interpolated from raw contours and used to define points along the SAM surface in the *xy* plane. SAM surface points were passed to the statistical software R (http://www.r-project.org/) and analysed by polynomial regression to the standard form of the parabolic equation:


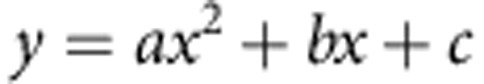


The coefficient 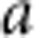 was taken as the shape-defining model factor and area was estimated by the equation: area=4/3(heights × radius), where height and radius were collected as below. Estimated area was compared with measured area collected by the ImageJ freehand selection tool.

For high-throughput analysis of SAM morphology, custom ImageJ macros and python scripts were used to process 1,186 DIC images of 14-day-old seedling SAMs from 369 inbred maize varieties. Using the point selection tool in ImageJ, we collected height (*h*) from the SAM apex and parabola radius (*r*) from the P1 notch from each image. From these primary measures, we calculated the following: height/radius=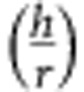, diameter=(2*r*), area=
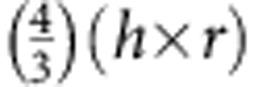
, volume=
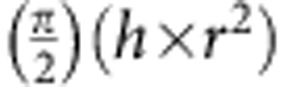
, parabolic standard form coefficient *a=*
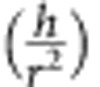
, SAM surface area=
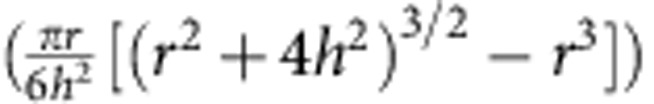
 and arc length=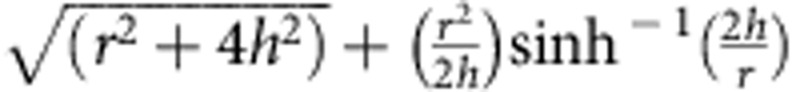. To account for germination differences in some inbred lines, best linear unbiased predictors (BLUPs) were calculated for all measures using SAS (http://www.sas.com/) and the nlme R package. BLUPs were used for GWAS, and phenotypes were reported in BLUP+intercept form.

For alignment of *in situ* hybridization serial sections, DIC images of RNA *in situ* hybridization slides were imported into ImageJ, placed in sequential order by morphological cues and aligned using the TrakEM2 package (http://fiji.sc/TrakEM2).

Images of fluorescent SAM nuclei were preprocessed in ImageJ for cell counting and size estimation using the freehand selection tool to remove cells outside the SAM. SAM images were analysed with a standard pipeline in CellProfiler^35^.

### SNP matrix generation

RNA for RNAseq analysis was extracted from SAM-enriched apices of 14-day-old seedlings and sequenced using Illumina HiSeq2000 instruments. The nucleotides of each raw read were scanned for low quality bases^63^. Bases with PHRED quality values<15 (out of 40)^64^, that is, error rates of ≤3%, were removed. Each read was examined in two phases. In the first phase, reads were scanned starting at each end and nucleotides with quality values lower than the threshold were removed. The remaining nucleotides were then scanned using overlapping windows of 10 bp and sequences beyond the last window with average quality value less than the specified threshold were truncated. Trimmed reads were aligned to the Maize B73 RefGen_v2 genome using GSNAP^26^,^65^,^66^. To obtain confidently mapped reads, reads were retained if they mapped uniquely in the genome, allowing two or less mismatches every 36 bp and fewer than five bases for every 75 bp in read length as unaligned ‘tails'. The coordinates of confident and single (unique) alignments that passed our filtering criteria were used for SNP discovery. Polymorphisms at each potential SNP site were examined and putative homozygous SNPs were identified using the following criteria after ignoring the first and last three aligned bases of each read. Before being used to call a SNP, a polymorphic base was required to have a PHRED base quality value of at least 20 (<1% error rate), and at least five unique reads must support the SNP call.

For genomic SNP calling, we used data from 2,815 US inbreds, including our 369 inbreds, which were genotyped at ∼700,000 SNP sites by sequencing^25^. The original data set was downloaded from Panzea (http://panzea.org). For accessions that were sequenced multiple times, we scored the consensus allele for each site. Alleles with conflicting records were scored as missing.

For SNP quality control, after merging RNASeq and genomic SNPs, polymorphisms with minor allele frequency <1% or missing in over 60% of inbreds were excluded from further GWAS analysis.

### Mixed-model GWAS

The analysis was performed on SAM volume BLUP data with a compressed mixed linear model^67^ implemented in the GAPIT R package (Version 3.55) by selecting the best model from PCA covariates and Kinship matrix^68^.

### *In situ* RNA hybridization

RNA *in situ* hybridization analyses were carried out as described in the study by Jackson^69^ with modifications as in the study by Johnston *et al*.^60^: FAA-fixed tissue was dehydrated and transferred to paraffin wax in preparation for sectioning. Longitudinal sections through the SAM were adhered to slides overnight, paraffin stripped off, rehydrated and treated by with Proteinase K in preparation for incubation with a DIG-labelled RNA probe. After overnight incubation at 50 °C with the *ZmLAX2*-specific probe, slides were rinsed several times in SSC, treated with RNase H to remove excess probe and incubated with an anti-DIG alkaline phosphatase (AP) conjugated Fab-fragment serum at 4 °C overnight (Roche Diagnostics, IN, USA). Transcript accumulation was visualized by incubating overnight at room temperature in a BCIP/NPT AP substrate (Roche Diagnostics).

SAM tissue from the following genotypes was examined: small SAM *ZmLAX2*-*COM* genotypes—CML322, B104, B57, NC314; large SAM *ZmLAX2*-*COM* genotypes—F42, CS405, NC324, LP5; large SAM *ZmLAX2*-*ALT* genotypes—ND246, Co255. We constructed an antisense probe to GRMZM2G129413 (*ZmLAX2*) using 1 kb of sequence from the last exon and 3′ untranslated region of inbred B73 cDNA, using primers oSL33 (5′- TCTATATCATCCCGGCGCTC -3′) and oSL38 (5′- TAACTTGCACCTTTGCTGCG -3′).

### Gene model annotation

Candidate genes model entries were queried on MaizeGDB (www.maizegdb.org) for classical names and best sequence homologues in *A. thaliana* and *O. sativa*. Genes without classical names were queried against a maximum likelihood protein sequence tree (http://ensembl.gramene.org). Protein domains were determined by SMART (http://smart.embl-heidelberg.de/).

### Field measurements

Stem diameter and node count measurements were collected in Summer 2014 at the Musgrave Research Farm (Aurora, NY). Measurements were gathered from 3 post-anthesis individuals from 10-kernel families of the 369 inbred varieties used above. The highest ear on the maize plant was designated the ‘primary ear.' The primary ear is clonally related to the node, internode and leaf on the opposite side of the stem, above its own point of insertion at maturity^70^. Stem diameter was collected from the widest diameter measured at the midpoint between nodes for: the clonally related internode above the primary ear, the internode at the point of insertion of the primary ear and internode below the point of insertion of the primary ear using a Fowler-Sylvac Digital Caliper Kit (Serialio.com, Cedar Park, TX). Above-ground nodes were scored and counted as a proxy for total leaf count.

### Statistical analysis and plotting

Descriptive statistical analysis, *t*-tests, one-way analysis of variance (ANOVA) and two-way ANOVA were carried out using core R packages. Correlation analyses were carried out using the PerformanceAnalytics R package. All correlations report Pearson's product-moment *r* and were evaluated for statistical significance with the Fisher transformation. Additional adult phenotype data for correlation analyses were collected from published data sets^24^.

## Additional information

**Accession codes:** Transcriptomic data are available through the NCBI sequence read archive (SRA) accession SRP055871.

**How to cite this article:** Leiboff, S. *et al*. Genetic control of morphometric diversity in the maize shoot apical meristem. *Nat. Commun.* 6:8974 doi: 10.1038/ncomms9974 (2015).

## Supplementary Material

Supplementary Figures Supplementary Figures 1-2

Supplementary Data 1 Unsummarized SAM image quantification. Direct measures of height and radius were used to estimate additional phenotypes based on a parabolic model.

Supplementary Data 2 Best Linear Unbiased Predictors (BLUPs) + coefficient for all genotypes examined. BLUPs model and account for batch effects and missing data between growth periods.

Supplementary Data 3 SAM BLUPs and adult phenotypes

Supplementary Data 4 Significant trait associated SNP (TAS) information identified by SAM volume GWAS.

Supplementary Data 5 TAS allele information.

Supplementary Data 6 SAM master regulatory gene SNPs. Known SAM master regulatory genes contain coding-region polymorphisms in this panel
