# Supplementary material for: Genetic control of morphometric diversity in the maize shoot apical meristem
Source: Nat Commun. 2015 Nov 20;6:8974. doi: 10.1038/ncomms9974 (PMC4673881; doi:10.1038/ncomms9974)
Supplement: Supplementary Figures — 1-2 [file ncomms9974-s1.pdf]

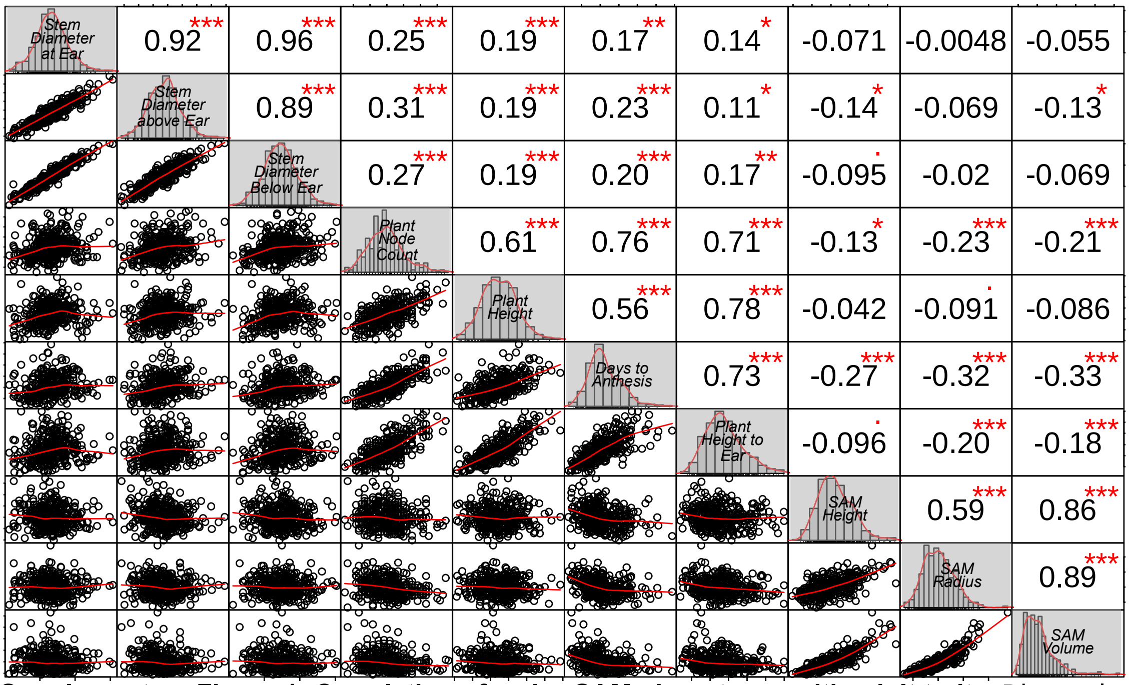

Supplementary Figure 1: Correlation of maize SAM phenotypes with adult traits. Diagonal plots show histogram of each variable with density kernel in red. Numbers above diagonal show Pearson's r between variables. Scatterplots below diagonal show XY relationship between variables with loess curves fit in red. Fisher transformation p-value: *** 0.001, ** 0.01, * 0.05, . 0.1.

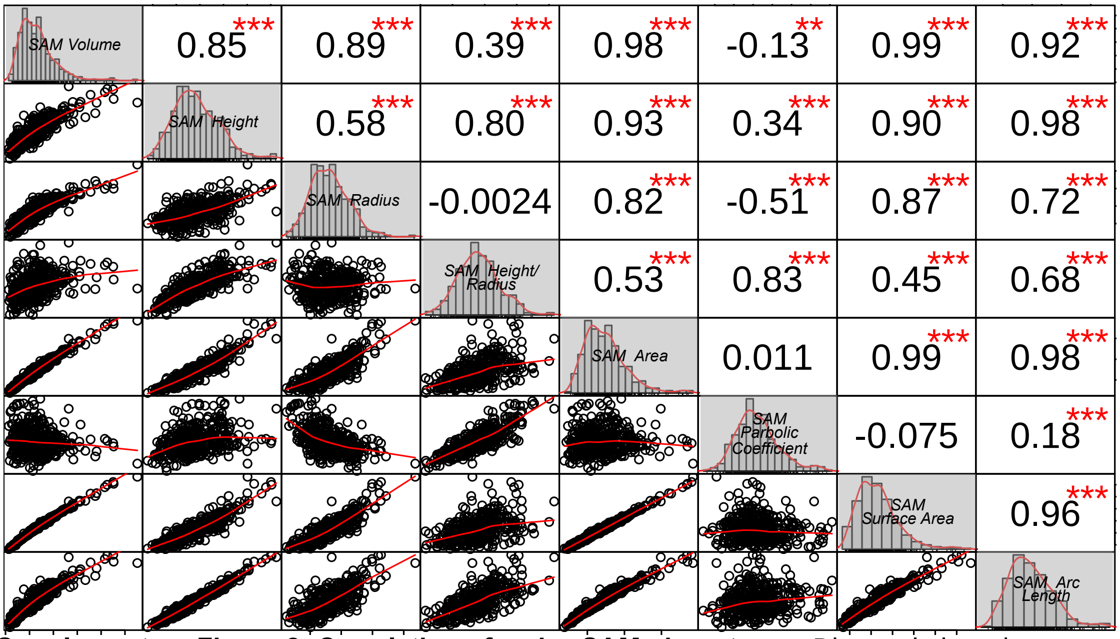

Supplementary Figure 2: Correlation of maize SAM phenotypes. Diagonal plots show histogram of each variable with density kernel in red. Numbers above diagonal show Pearson's r between variables. Scatterplots below diagonal show XY relationship between variables with loess curves fit in red. Fisher transformation p-value: *** 0.001, ** 0.01, * 0.05, . 0.1.
